# Leveraging electronic health records for atrial fibrillation cohort generation

**DOI:** 10.1007/s13755-025-00415-w

**Published:** 2026-01-07

**Authors:** Ane G. Domingo-Aldama, Marcos Merino Prado, Alain García-Olea, Josu Goikoetxea, Koldo Gojenola, Aitziber Atutxa

**Affiliations:** 1 University of the Basque Country UPV/EHU, 48013 Bilbao, Vizcaya Spain; 2 Basurto University Hospital, 48013 Bilbao, Vizcaya Spain

**Keywords:** Cohort selection, Eligibility screening, Large language models, Electronic health records, Atrial fibrillation progression

## Abstract

**Purpose:**

Cohort selection and eligibility screening are critical in clinical research, especially in trials where manual patient matching remains a major bottleneck. This study investigates the use of Natural Language Processing and Large Language Models (LLMs) in two real use cases, namely Atrial Fibrillation (AF) progression and Hearth Failure (HF) decompensation, within a non-English clinical context. We specifically address the following research questions: (1) Can discharge reports and NLP support cohort selection? (2) Can LLMs effectively model longitudinal patient trajectories and temporal reasoning? (3) Do general-purpose or domain-adapted LLMs outperform rule-based baselines for this task? (4) Compared to large foundation models, do small-scale LLMs offer similar performance?

**Methods:**

A dataset of 212 patients was manually annotated for AF progression using discharge reports. Two strategies were evaluated: (1) an adapted rule-based pipeline and (2) zero-shot open-source LLMs with varying prompt structures. To assess generalizability, an additional dataset of 100 patients was annotated for HF decompensation.

**Results:**

The adapted rule-based approach achieved the highest accuracy (0.82), but LLMs with task-division prompts performed comparably (up to 0.79), requiring significantly less manual effort. The medium-sized general-domain *gemma-3* model outperformed others.

**Conclusions:**

(1) Discharge reports are a valuable resource for automatic cohort selection, with both the rule-based method and LLMs showing promising results. (2) While LLMs struggled with long-context inputs, they handled temporal reasoning well when explicit dates were provided. (3) Larger models did not always outperform smaller ones, (4) prompt language strongly influenced performance, and medical model variants were not consistently superior.

## Introduction

Accurate cohort selection plays a central role in medical research, particularly in clinical trials [1–3], epidemiological studies [4, 5], public health surveillance chronic diseases [6, 7] or early diagnosis and risk studies [8], as it determines the population under investigation and directly impacts the reliability, generalizability, and statistical power of the results. High-quality cohort selection ensures that patient groups are appropriately matched to study criteria, while minimizing bias. However, identifying suitable patients often involves time-consuming manual review of clinical documents, requiring domain expertise to interpret unstructured narratives, assess eligibility criteria, and resolve ambiguities in medical terminology. This labour-intensive process not only delays research timelines but also introduces variability and potential human error [1, 2].

A commonly adopted alternative is the use of structured data manually coded from electronic health records (EHRs). However, the process of coding structured data is prone to human error and often results in incomplete or missing information [9, 10]. Despite efforts to standardize medical coding with systems like ICD-10,^1^ OPCS,^2^ and SNOMED,^3^ there are no universal rules for how detailed clinical documentation coding should be. Coding practices differ significantly between countries and even within institutions.

Consequently, the growing interest in Natural Language Processing (NLP) has led to the development of numerous approaches for cohort selection, initially based on rule-based systems and traditional machine learning techniques [11, 12]. However, these methods often suffer from limited generalizability, as their performance heavily depends on the quality and coverage of manually crafted rules. Furthermore, adapting such systems to new clinical domains or institutions typically requires significant reengineering efforts. In light of recent advances in text understanding and generation, Large Language Models (LLMs) have emerged as promising tools for automating cohort selection [13–15].

**Fig. 1:**
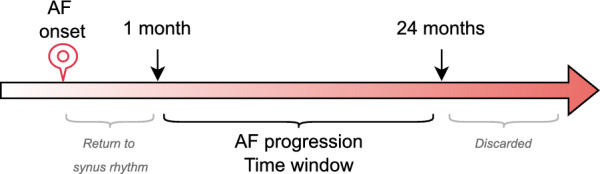
Time-window for AF progression. AF progression (recurrence or permanence) is evaluated between 1 month and 2 years after the initial onset, the return to sinus rhythm is assessed starting from 1 day after the debut episode

This article presents a comparative analysis of various approaches to cohort selection in the context of Atrial Fibrillation (AF) progression. The study focuses on leveraging free-text discharge reports of patients, combined with a variety of NLP techniques, to identify the recurrence or permanence^4^ occurring between 1 month and 2 years following the initial diagnosis (see Fig. 1).

Moreover, to ensure the generalizability to other chronic conditions, the optimal strategy is evaluated for the detection of heart failure (HF) decompensation. Heart failure decompensation refers to a worsening of previously stable heart failure, where the heart is suddenly unable to pump enough blood to meet the body’s needs. Consequently, the identification of HF decompensation requires a debut episode of the disease and a new episode of HF from 1 week to 2 years after the initial episode.

The identification of AF progression and HF decompensation is clinically meaningful, as it enables the development of predictive models to anticipate disease trajectory. Atrial fibrillation is the most prevalent cardiac arrhythmia worldwide, while heart failure is the leading cause of cardiovascular-related hospitalizations in Spain. In both conditions, treatment strategies and prognosis depend heavily on accurately assessing the risk of progression or decompensation. Consequently, predictive models capable of identifying patients at increased risk of AF progression or HF decompensation represent valuable decision-support tools, with the potential to improve clinical management, optimize healthcare resources, and reduce preventable hospital readmissions.

To this end, the research is guided by the following core questions:

- **RQ1**: Can discharge reports, in combination with NLP techniques, be effectively used for cohort selection?
- **RQ2**: Which approach among rule-based methods and LLMs is most effective for automatic cohort selection?
- **RQ3**: Can LLMs effectively process long real clinical narratives, longitudinal information and reason over temporal relationships between events to support cohort selection?
- **RQ4**: Compared to proprietary large foundation models, do small-scale open-source LLMs offer similar performance regardless of their domain specificity?

Challenges such as *n2c2* and the *TREC Clinical Trials Track* have proposed tasks related to automatic cohort selection. However, these tasks typically focus on identifying diseases or symptoms within isolated, short, and synthetically created clinical notes, which limits their complexity compared to real-world scenarios. The present research addresses two real complex and temporally dependent clinical use-cases (AF progression and HF decompensation). Both conditions require not only the identification of an initial episode, but also the recognition of a subsequent episode occurring within a specific time window. Temporal reasoning is specially challenging as addressed by several works [16–18].

Compared to prior work, this study advances the state of the art in several key ways. First, while most existing approaches rely on a single clinical note or limited temporal context, we propose a methodology that leverages the patient’s clinical history, incorporating all available discharge reports. This allows for more accurate patient-trial matching by explicitly modelling temporal reasoning across multiple documents, a known challenge in eligibility screening. Moreover, although the present work focuses on AF progression and HF decompensation, the proposed approach is broadly applicable to other conditions where cohort selection relies on the interpretation of longitudinal patient data rather than isolated encounters, for instance, Relapsing-Remitting Multiple Sclerosis or Chronic Obstructive Pulmonary Disease.

Second, the study evaluates both LLMs and rule-based systems on their ability to process long-context inputs and reason over time-dependent clinical events, bridging a gap in current research that often overlooks longitudinal context or evaluates models on simplified, static inputs. To the best of our knowledge, no prior research has directly compared rule-based systems with LLM-based models in the cohort generation field. Therefore, this work opens a new avenue for understanding the strengths and limitations of the latter in this domain.

Finally, we explore the performance of open-source, low-resource LLMs in a non-English clinical setting, addressing two major barriers to real-world deployment: the computational cost of LLMs and the language gap in clinical NLP research. This makes our findings directly applicable to resource-constrained healthcare environments where scalability, efficiency, and multilingual support are critical.

## Related work

Automated cohort generation has traditionally relied on structured data and manually designed queries [19–21]. This task can be framed as an information retrieval problem, and recent advances in NLP have led to a growing body of research focused on automating patient cohort selection.

The TREC Clinical Trials track of 2021 [22] and 2022 [23] are a clear example of recent advancements in cohort selection. The TREC Clinical Trials track focused on the task of cohort retrieval: given a brief natural-language clinical description (5–10 sentences) of a patient, systems had to retrieve relevant clinical trial protocols from *ClinicalTrials.gov* that matched the patient’s eligibility. Although the TREC task centered on retrieving protocols for trial recruitment, it shares fundamental similarities with our objective: generating clinically coherent patient cohorts from unstructured narratives. Both efforts involve interpreting clinical narratives, managing missing or ambiguous data, and matching against predefined clinical criteria.

Prior to the advent of LLM-based methods, cohort selection from EHRs relied on rule-based NLP systems, which used handcrafted lexicons and pattern-matching logic to flag eligible patients or Machine Learning Systems that required an intensive feature engineering process. Rule-based systems achieved respectable performance in controlled shared-task settings but required extensive domain expertise to develop and maintain, and they often struggled to generalize across institutions or criteria [11]. Consequently, hybrid approaches have emerged to surpass these limitations including the mix of rules with machine learning techniques [24–26].

Several recent approaches leverage fine-tuned discriminative language models for cohort selection using EHRs [27–29]. Although several transformer-based models are capable of modeling longitudinal EHR data [30–33], cohort selection using these models remains an open research area. Additionally, the reliance on substantial labelled training data presents a major hurdle in settings where annotations are costly or scarce. Even with abundant training examples, these models exhibit known weaknesses in processing long contexts and in handling temporal reasoning, and in many cases they fail to exceed the performance achieved by traditional retrieval models in various benchmarks [23].

Recent studies have used generative LLM, for example, Guan et al. [34] introduced CohortGPT, a framework that combines GPT-based language models with domain knowledge and chain-of-thought prompting to enhance participant recruitment. In this regard, en Tai and Tannier [13] evaluated LLMs in the cohort selection challenge of the n2c2 clinical trial. They found that while LLMs performed well on general eligibility rules, they struggled with fine-grained logic and temporal conditions, revealing the need for further development to ensure clinical reliability. In another work, Rybinski et al. [14] introduce a multi-stage patient-to-trial matching pipeline, evaluated on the TREC Clinical Trials benchmarks. The pipeline includes traditional retrieval components (like BM25 and transformer-based rankers) followed by LLM-based query formulation and fine-tuned re-ranking, boosting performance in key metrics such as Normalized Discounted Cumulative Gain (NDCG) and precision.

Fewer studies have tackled the specific challenge of identifying AF progression, particularly from unstructured clinical narratives. Zheng et al. [35] developed a rule-based NLP pipeline to extract recurrent AF events from discharge summaries and found that it significantly outperformed code-based algorithms, underlining the value of narrative clinical text for this task. Similarly, Feng et al. [36] explored the use of GPT-3.5 and GPT-4 for detecting arrhythmia progression (more concretely, AF recurrence). Through prompt engineering and the inclusion of rationale-based responses, they substantially improved the accuracy of recurrence detection, surpassing both traditional NLP and fine-tuned BERT models.

Two key technical challenges in identifying eligible patients to study AF progression from clinical narratives are reasoning over long contexts and understanding temporal relationships between events. Although the context window of LLMs continues to expand, several studies have shown that their true capacity for long-context comprehension remains limited [37, 38]. To address this, techniques such as task decomposition and prompt engineering have been proposed. For example, Zhang et al. [39] introduced the Chain-of-Agents framework, in which multiple LLMs collaborate by dividing complex tasks into smaller subtasks and then recombining the results. This method demonstrated significant improvements in handling extended input while preserving coherence, suggesting that task division is an effective strategy for overcoming context length limitations in LLMs.

In parallel, other research has focused on evaluating LMs on temporal reasoning tasks [40–42]. The general results show that they often struggle with complex temporal constructs such as interval overlap, conditional dependencies and arithmetic operations.

## Dataset

The present research has been possible thanks to the following two resources kindly provided by the Basque Public Healthcare System (Osakidetza):

- *Discharge reports in Spanish* a pool of $1.2\times 10^{6}$ discharge reports dating from 2015 to 2020.
- *Codified structured data from the Osakidetza Business Intelligence (OBI) system* clinical data for each patient, encoded by healthcare professionals using standardized coding systems and stored in a Business Intelligence platform. This data is stored in tabular format (patient, feature, value, date) and includes clinical information such as each patient’s AF debut episode and the corresponding date.

### AF progression dataset

**Fig. 2:**
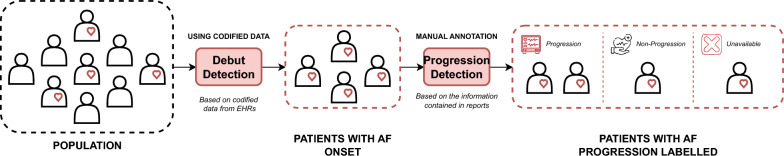
Manual annotation process of the dataset. The initial filtering for AF debut is performed using information from the OBI system, followed by the manual annotation of AF progression

We identified a subset of 212 patients with a documented AF onset^5^ in the OBI system. The progression status for each patient was manually annotated by two annotators by leveraging their discharge reports, following a set of guidelines developed in collaboration with a cardiologist from the BioBizkaia research institute (see “Appendix 1”). The annotation protocol categorized each patient into one of three classes based on their progression status within the period ranging from 1 month to 2 years after the initial AF episode (see Fig. 2):

- **Progression** (1): the patient has a documented recurrence or permanence of AF between 1 month and 2 years after the onset.
- **Non-progression** (0): the patient returned to sinus rhythm, with no evidence of AF recurrence or permanence at least 1 day after the onset.
- **Unknown** ($-1$): the discharge reports lacked sufficient information to determine the progression status.

Annotating AF progression status is a challenging task that requires both clinical expertise and contextual understanding. It involves identifying varied medical expressions referring to AF episodes, reasoning over the patient’s full clinical history, and interpreting temporal relationships to determine whether the patient returned to sinus rhythm or experienced recurrence or permanence. The complexity of these terms and the need for longitudinal analysis make the task particularly demanding.

**Fig. 3:**
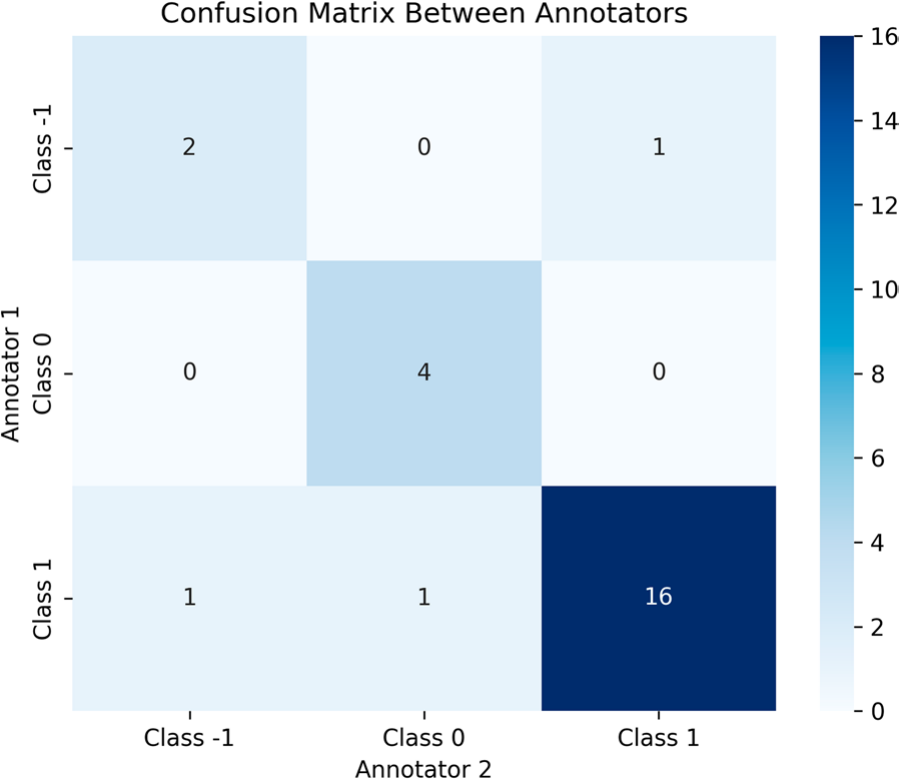
Confusion matrix showing the level of agreement between annotators based on their respective labels. * Class 1* represents AF progression (recurrence or permanence), *Class 0 *indicates non-progression (return to sinus rhythm), and $\textit{Class -1}$ includes cases with insufficient information to determine progression status

The obtained inter-tagger agreement (ITA) calculated by leveraging the Cohen’s kappa coefficient is 0.741 (see Fig. 3). Based on the Landis and Koch table on interpretation of kappa values [43], this means a good agreement between annotators. The final dataset characteristics are available in Table 1.

**Table 1:** Overview of the dataset

	Size	Progr.	Non-Progr.	Unknown
*Dev*	104	50	17	37
*Test*	108	41	25	42

The second column indicates the total number of patients in each subset. The third, fourth, and fifth columns show the number of patients in each class: Progr. (AF recurrence or permanence), Non-Progr. (return to sinus rhythm), and Unknown (insufficient information)

### HF decompensation dataset

We manually annotated a subset of 100 patients with a codified HF first episode in the OBI system. Four annotators following a set of guidelines (see “Appendix 1”) generated by the same cardiologist and by leveraging the discharge reports of each patient manually annotated the decompensation status of each patient into the following three classes:

- **Decompensation** (1): the patient has a documented initial episode of HF, followed by a documented episode of HF decompensation occurring between 1 week and 2 years after the debut.
- **Non-Decompensation** (0): the patient has a documented initial episode of HF but no subsequent HF episodes reported.
- **No-debut** ($-1$): the patient has no documented initial episode of HF in any report.

Once again, the calculated ITA, measured using Cohen’s kappa coefficient, is 0.88, this reflects a high level of agreement between annotators. The characteristics of the final dataset are summarized in Table 2.

**Table 2:** Overview of the HF decompensation dataset

	Size	Decomp.	Non-Decomp.	No-debut
*Test*	100	32	25	43

The second column indicates the total number of patients in each subset. The third, fourth, and fifth columns show the number of patients in each class: Decomp. (HF decompensation), Non-Decomp. (no new episode of HF), and No-debut (Decompensation episode found with no documented debut episode)

## Experimental setup

This section describes the experimental framework of the study. We first present the AF progression experiments, beginning with the adapted rule-based methodology and its main components (“Adapted rule-based method” section), followed by a detailed description of the LLM-based approach and the different prompt strategies evaluated (“LLM-based method” section). Finally, we describe the HF decompensation experiment (“Experiments for HF decompensation” section), which applies the best LLM-based strategy.

### Adapted rule-based method

**Fig. 4:**
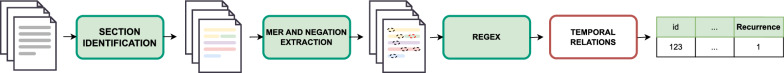
Overview of the rule-based approach. The pipeline includes a section identification module, a medical entity recognition (MER) and negation detection module, advanced regular expressions, and temporal management of clinical events to determine AF progression status

The adapted rule-based cohort selection method follows a three-step pipeline (see Fig. 4):

- The process begins with a section identification tool [44] that segments each discharge report into clinically meaningful components, allowing the system to distinguish between past and present events, as well as between family history and the patient’s clinical status.
- A medical entity recognition (MER) and negation detection module [45] is then applied to extract important clinical concepts—such as AF episodes and procedures—and determine whether they are negated in context.
- Regular expressions are used to identify specific mentions of AF episodes and returns to sinus rhythm within the extracted content.
- Finally, using the dates associated with each report and the identified clinical events, the system determines the AF progression label based on the temporal criteria of the task—specifically, whether a new AF episode occurs between 1 month and 2 years after the initial diagnosis.

Although this rule-based approach is based primarily on regular expressions, it is not a pure rule-based approach because it also introduces two previous discriminative LM modules based on the Flair architecture [46] that help with the limitations encountered in rule-based solutions, which are negations and generalizability. Both of them are partially surpassed thanks to the negation extraction and MER tool.

### LLM-based method

The second approach explored in this study is the use of LLMs for automatic cohort selection. As discussed in “Related work” section, LLMs often face limitations when dealing with long-context inputs and temporal reasoning–both of which are critical challenges in the task of detecting AF progression. To address these issues, the experiments presented in this subsection investigate a range of strategies, from simpler to more advanced, all relying exclusively on LLMs. The goal is to assess their effectiveness in handling complex temporal and contextual dependencies inherent in clinical narratives.

The LLMs selected for this experimentation comprise a diverse set of architectures, Spanish knowledge level and, in some cases, additional clinical domain knowledge (see Table 3).

**Table 3:** LLMs selected for the experiments

Model name	Citation	Architecture	Clinical
*gemma-3-12b-it*	GemmaTeam [47]	Gemma 3	No
*gemma-3-27b-it*	GemmaTeam [47]	Gemma 3	No
*medgemma-27b-text-it*	GemmaTeam [48]	Gemma 3	Yes
*Llama-3.1-8B-It*	AI@Meta [49]	LLaMA 3.1	No
*Llama3.1-Aloe-Beta-8B*	Gururajan et al. [50]	LLaMA 3.1	Yes
*Mistral-7B-Instruct-v0.3*	Jiang et al. [51]	Mistral	No

The table includes each model’s name and citation, its underlying architecture, and whether it incorporates clinical domain knowledge in its pretraining

In total, three main prompt strategies were explored, one of which includes four adaptations for temporal management. Due to context length limitations, all prompts were designed for zero-shot inference. Each strategy was tested in both English and Spanish to assess the impact of the prompt language on model performance. All the prompts are available in “Appendix 2”.

In addition, “Preliminary experiments with fine-tuning strategy” section presents a fine-tuned LLM strategy to evaluate model performance when specifically trained for the task of AF progression detection.

All prompt engineering was conducted using the development set, with final evaluations carried out on the test set.

#### Concatenation strategy (baseline)

The baseline experiment involved concatenating all available discharge reports for each patient into a single input. The objective of this approach is to evaluate the ability of LLMs to handle long-context inputs and reason over temporal relationships without any external preprocessing or assistance.

#### Summarization strategy

**Fig. 5:**
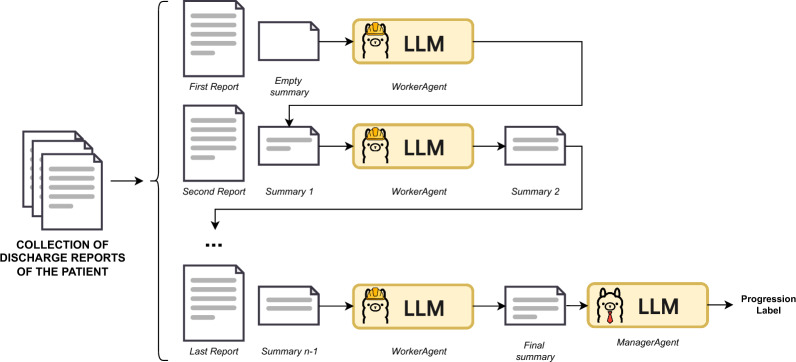
Summarization Strategy Each discharge report is summarized in conjunction with the previous summary, and the final decision is made based on the complete accumulated summary

Building on the approach described in Zhang et al. [39], this second method employs a multi-agent framework to perform the classification task. In this setup, a dedicated agent, referred to as the *WorkerAgent*, is assigned to each discharge report. The agent summarizes the content of the report and incorporates the summary of the preceding clinical history—starting with an empty summary for the first report (see Fig. 5).

The summarization process is explicitly guided to extract only the information relevant for determining AF progression, such as electrocardiogram results, AF episodes, cardioversion procedures, and ablations. Once the complete clinical trajectory has been summarized, a *ManagerAgent* reviews the final summary and issues the classification decision regarding AF progression status. The possible outputs are ‘Yes’ (recurrence or permanence), ‘No’ (no progression), or ‘Unknown’ (insufficient information).

#### Onset-guided strategy

**Fig. 6:**
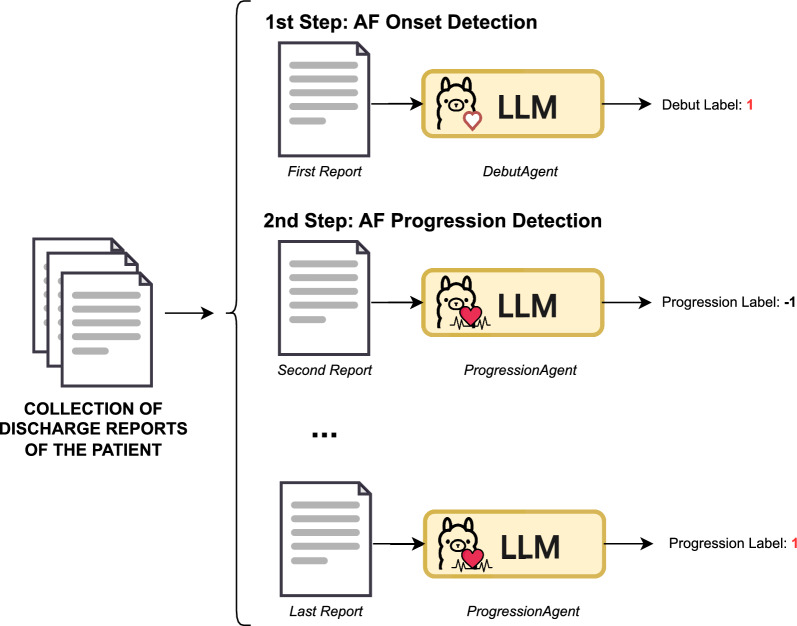
Onset-Guided Strategy. The process is divided into two stages: first, identifying the AF onset, and second, determining the progression status

To address the challenges posed by long-context inputs in modeling the full clinical history of a patient, this approach decomposes the task into two sequential substeps (see Fig. 6): first, the identification of the AF onset episode, and second, the classification of subsequent events as either indicative of AF progression, sinus rhythm, or neither.

In the first step, AF onset identification is treated as a binary classification problem. Each discharge report is independently evaluated, with the model predicting ‘Yes’ if the report contains evidence of the initial AF diagnosis, or ‘No’ otherwise. The search for AF progression does not begin until the debut episode has been identified.

Once the onset is located, the second step focuses on detecting AF progression. This is formulated as a three-class classification task with possible outcomes of ‘Yes’ (recurrence or permanence), ‘No’ (ECG returning to sinus rhythm), or ‘Unknown’ (insufficient information). Both the debut episode date and the content of the current discharge report are provided explicitly in the prompt as input to support temporal reasoning and contextual understanding necessary for an accurate decision.

To investigate how LLMs interpret and reason with dates, three different input formats were evaluated.

- **Numeric date format (** ***Num*)**: this format presents dates in a standardized numerical representation. *Example:* 2018-08-11.
- **Written date format (*NL*)**: dates are expressed in natural language, aiming to assess the model’s ability to comprehend temporal expressions in free-text form. *Example:* August 11, 2018.
- **Offset (*Dur*)**: instead of providing explicit dates, the duration in days between the AF debut and the current discharge report is calculated and included. This format isolates the temporal gap as a numerical input, enabling the model to directly consider the time elapsed since the debut event when determining progression. *Example:* Time since debut: 18 days.

#### Chronology-guided strategy

**Fig. 7:**
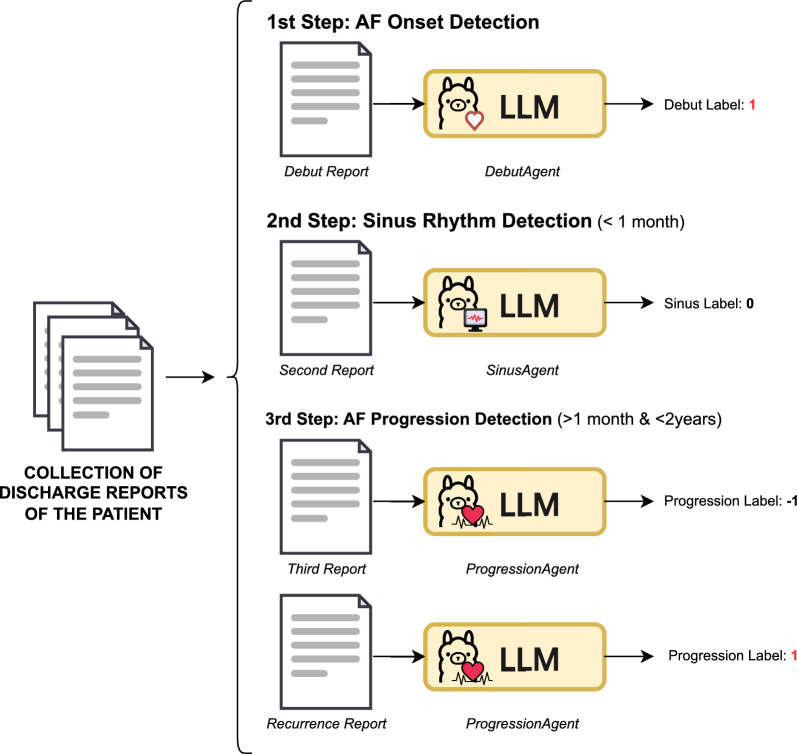
Chronology-Guided Strategy. The process is divided into three stages: first, identifying the AF onset, second, determining the return to sinus rhythm in reports within 1 month after the AF onset, and third, determining the progression status for reports between 1 month and 2 years after the AF onset

A final experiment was designed to explicitly assess the limitations imposed by the temporal reasoning requirements of this task. To do so, the classification process was divided into several subtasks, mirroring the structure of previous experiments but with stricter temporal constraints (see Fig. 7).

First, the AF debut episode is identified using the same prompt applied in the *Onset-Guided* strategy. Then, all discharge reports dated within 1 month after the debut are evaluated by the LLM to detect a possible return to sinus rhythm. Subsequently, reports falling between 1 month and 2 years after the debut are processed to determine AF progression, using the three-class classification scheme. Finally, any reports occurring more than 2 years after the AF debut are excluded.

This segmentation of the clinical timeline is feasible because each discharge report includes an associated date, allowing the data to be chronologically ordered, filtered, and analyzed based on its temporal relevance to the task.

#### Preliminary experiments with fine-tuning strategy

While the primary motivation of this study is rooted in real clinical scenarios with constrained resources, we also include a fine-tuning strategy using QLoRA as a point of comparison against the previously proposed zero-shot approaches.

Fine-tuning is applied to the best model–strategy combination identified in “Results and discussion” section. Specifically, we use a training subset of 250 clinical histories together with the development subset described in Table 1, enabling the LLMs to specialize in the cohort generation task for AF progression.

Given that the selected strategy follows a two-step design, we constructed separate datasets for AF onset identification and AF progression detection using the reports of each patient. In addition, 10% of the data was allocated to the evaluation set during training. The resulting dataset sizes for each step are reported in Table 4.

**Table 4:** Overview of the fine-tuning dataset

	Size	Positive	Negative	Unknown
*Onset*	698	349	349	–
*Progression*	624	176	75	373

The second column indicates the total number of patients in each subset. The third, fourth, and fifth columns show the number of patients in each class: Positive (AF debut/progression), Negative (no debut/sinus rhythm), and Unknown (insufficient information)

The different runs explored variations in learning rates as well as the use of balancing versus non-balancing of the progression detection dataset. Although this strategy could be further explored through more comprehensive hyperparameter optimization, such an analysis lies beyond the scope of this article, which deliberately considers constrained resources to reflect a realistic clinical scenario.

### Experiments for HF decompensation

The experiments on HF decompensation detection build upon the best strategy identified for the AF progression detection task, with the corresponding adaptations made to the prompts. As shown in the next section (“Results and discussion” section), the best performance for AF progression was achieved using the Onset-Guided strategy with numerical dates.

For HF decompensation, the first step involves detecting the debut episode. This step yields two possible labels: ‘Yes’ (a debut is identified), ‘No’ (no debut is identified). The second step applies to patients for whom a debut episode was identified. In this step, the debut report and subsequent reports are provided to the model to determine whether an HF decompensation occurred. The possible outcomes are ‘Yes’ (a decompensation episode is identified) or ‘No’ (no evidence of decompensation is found).

## Results and discussion

This section presents the main findings of the study. We first report the results of the AF progression detection task (see “Results for AF progression” section), analyzing the performance of both the rule-based and LLM-based strategies. Subsequently, we evaluate the generalizability of the proposed approach through its application to the HF decompensation detection task (see “Results for HF decompensation” section).

### Results for AF progression

The results presented in Table 5 are obtained using the test set while all the prompt engineering was performed using the development set. For the LLM-based models, the results count as errors all the answers where the instructions are not followed even when the correct answer might be inside the returned text, this decision aimed to evaluate the capacity of the models to follow instructions and format.

**Table 5:** Performance results for each strategy

Strategy	Model	Prompt language	Accuracy	F1-score	Format errors
Rule-based	–	–	***0.82***	***0.82***	–
Concatenation	*gemma-3-12b-it*	es	0.34	0.32	1
	*gemma-3-27b-it*	es	0.41	0.31	0
	*MedGemma-27b*	en	0.28	0.26	38
	*Llama-3.1-8B-It*	en	**0.49**	**0.46**	0
	*Llama3.1-Aloe-Beta-8B*	en	0.4	0.26	0
	*Mistral-7B-Instruct-v0.3*	es	0.26	0.20	12
Summarization	*gemma-3-12b-it*	es	0.59	0.58	0
	*gemma-3-27b-it*	es	**0.69**	**0.67**	0
	*MedGemma-27b*	en	0.63	0.62	0
	*Llama-3.1-8B-It*	en	0.59	0.58	0
	*Llama3.1-Aloe-Beta-8B*	es	0.49	0.49	0
	*Mistral-7B-Instruct-v0.3*	es	0.15	0.15	67
Onset-Guided + Num	*gemma-3-12b-it*	es	***0.79***	***0.79***	1
	*gemma-3-27b-it*	en	0.74	0.74	0
	*MedGemma-27b*	es	0.70	0.72	5
	*Llama-3.1-8B-It*	en	0.48	0.48	7
	*Llama3.1-Aloe-Beta-8B*	en	0.62	0.63	8
	*Mistral-7B-Instruct-v0.3*	en	0.24	0.33	68
Onset-Guided + NL	*gemma-3-12b-it*	es	**0.78**	**0.78**	1
	*gemma-3-27b-it*	en	0.74	0.74	0
	*MedGemma-27b*	es	0.70	0.72	5
	*Llama-3.1-8B-It*	en	0.48	0.47	6
	*Llama3.1-Aloe-Beta-8B*	en	0.59	0.61	8
	*Mistral-7B-Instruct-v0.3*	en	0.24	0.33	66
Onset-Guided + Dur	*gemma-3-12b-it*	en	**0.74**	**0.74**	0
	*gemma-3-27b-it*	en	0.74	0.74	0
	*MedGemma-27b*		0.67	0.68	5
	*Llama-3.1-8B-It*	en	0.50	0.47	3
	*Llama3.1-Aloe-Beta-8B*	en	0.55	0.57	8
	*Mistral-7B-Instruct-v0.3*	en	0.24	0.33	69
Chronology-Guided	*gemma-3-12b-it*	es	**0.77**	**0.77**	1
	*Llama-3.1-8B-It*	en	0.61	0.60	3
	*Llama3.1-Aloe-Beta-8B*	en	0.66	0.69	8
	*Mistral-7B-Instruct-v0.3*	en	0.18	0.28	98
Onset fine-tuning	*gemma-3-12b-it*	es	0.69	0.69	0

The best and second-best values within each strategy are highlighted in **bold** and underlined, respectively

While our rule-based pipeline achieves the highest absolute accuracy (0.82), LLMs paired with task-division prompting deliver nearly equivalent results (0.79) with far less upfront investment. By decomposing the problem into subtasks these models avoid the laborious development of regular expressions and the application of section identification and entity recognition tools. This reduction in preprocessing and human engineering makes LLM-based methods considerably more scalable and adaptable to new datasets or clinical conditions.

Across all evaluated LLMs, the *gemma-3-12B-it* model emerged as the most effective LLM for AF progression cohort selection across all the prompt strategies. Its strong performance reflects a balanced capability to process clinical prompts and consistently produce the required classification labels. However, other models demonstrated difficulties in accurately following the prescribed instructions. Many models (including domain-adapted ones) tended to return explanations rather than a simple label, forcing extensive prompt engineering to constrain outputs. Consequently, *gemma-3-12B-it* stood out by faithfully following label-only instructions.

The results show that increasing the size of the model did not guarantee better results. The 27B-parameter *gemma-3-27B* underperformed relative to its 12B counterpart. It also required significantly greater compute resources. Therefore, in practical clinical settings, where efficiency, clarity, and resource constraints are paramount, the smaller *gemma-3-12B-it* offers the most realistic and reliable solution.

Regarding the clinical LLMs, although Aloe-Beta consistently outperforms its general-purpose counterpart Llama3.1, MedGemma shows clear limitations in adherence to instructions. Specifically, it frequently provides explanatory text alongside its predictions, rather than returning the required label only. This behavior poses a challenge for tasks where concise, standardized outputs are necessary–particularly in scenarios that involve automatic post-processing of model responses. However, even in cases where the output format was not a limitation, MedGemma did not outperform its generic counterpart, although it included clinical knowledge and being a larger model than the *gemma-3-12B-it* (12 Billion parameters versus 27 Billion parameters).

When presented with the full concatenated clinical history, all LLMs suffered from the classic “long-context” limitation. The *Concatenation* strategy, which simply merged every discharge report, saw performance drop substantially, confirming that unstructured, extensive inputs still overwhelm current architectures.

The format in which temporal information is provided also proved important. Models performed best when dates appeared in standardized numeric (yyyy-mm-dd) or written formats, but obtained worse results when asked to reason over relative durations in days. This suggests that, although LLMs can parse and compare explicit dates, their inherent arithmetic abilities are insufficient for reliable duration calculations.

**Fig. 8:**
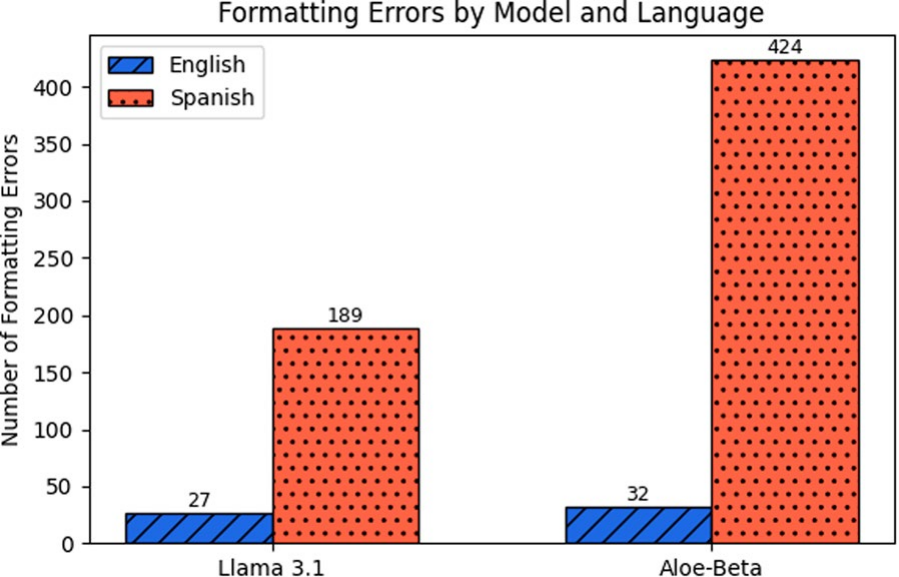
Total format errors across experiments by prompt language in Llama models. Each bar shows the total number of format errors made by the Llama 3.1 (left) and Aloe-Beta (right) models across all strategies, with blue (scratched) representing English prompts and red (dotted) representing Spanish prompts

**Fig. 9:**
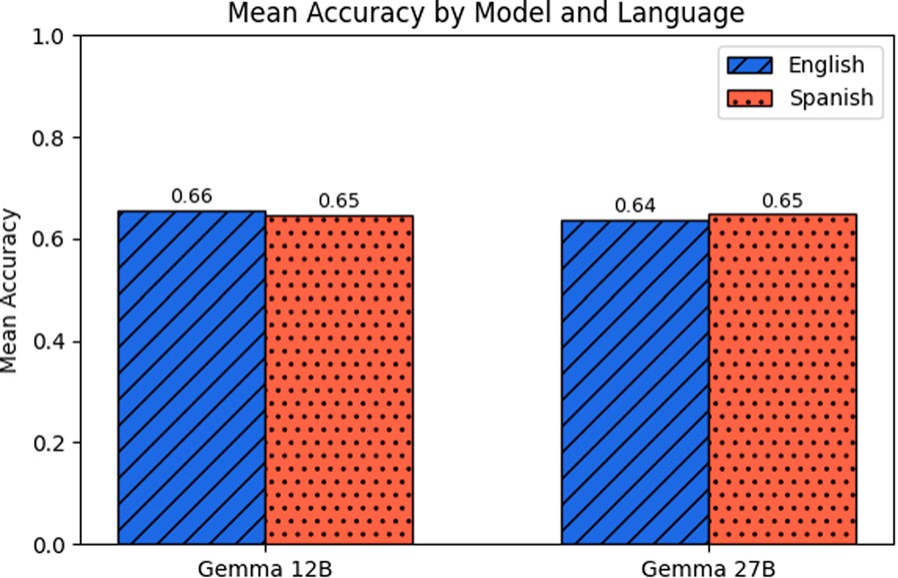
Mean accuracy across experiments for each prompt language in Gemma models. Each bar represents the average accuracy achieved by the *Gemma-12B* (left) and *Gemma-27B* (right) models across all strategies, with blue (scratched) indicating English prompts and red (dotted) indicating Spanish prompts

Regarding prompt language, models based on the LLaMA architecture generally performed better with prompts written in English, largely due to difficulties in following instructions when prompted in Spanish (see Fig. 8). A similar issue was observed with the MedGemma model, which tended to include reasoning in its responses when prompted in Spanish, despite being explicitly instructed to return only the label. The Mistral models exhibited instruction-following issues in both languages, although the problem was more pronounced in Spanish. In contrast, the Gemma models did not exhibit a consistent preference for either prompt language when evaluating mean accuracy across strategies (see Fig. 9). However, when excluding the summarization-based experiments, the mid-sized model (*gemma-3-12b-it*) showed a clear preference for Spanish prompts. This suggests that for classification tasks, Spanish prompts may be more effective, while English appears to be favored in more complex text generation tasks such as summarization. Interestingly, this language preference shift was not observed in the larger *gemma-3-27b* model, which showed no significant language bias across tasks.

The fine-tuned version of *gemma3-12b-it* with the Onset-Guided strategy yielded poorer results compared to the zero-shot approach. A possible explanation is that fine-tuning may reduce the model’s ability to generalize, particularly in long-context tasks, which are known to present significant limitations. However a further analysis of the results and the optimization parameters of the fine-tuned version could be explored in the future.

#### Further analysis of the best LLM strategy

To further analyze the performance and robustness of the proposed solution, the best pair of LLM and strategy was selected (*gemma-3-12b-it* and Onset-Guided + Num with Spanish prompts) and will be discussed in the following subsections.

***Error analysis***

**Fig. 10:**
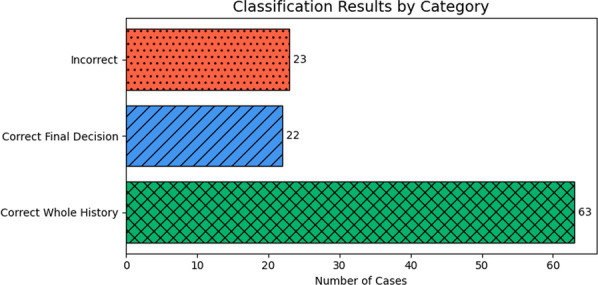
Prediction outcomes analysis. Red (dotted) indicates incorrect final predictions, blue (scratched) represents correct final predictions with intermediate errors, and green (crossed) denotes fully correct predictions at every decision point

Firstly, a manual revision of each prediction and error was performed. Figure 10 presents the outcomes of a step-by-step analysis of model predictions across patient clinical histories. The categories are as follows:

- *Correct Whole History (53 cases)* these are cases in which the model made accurate predictions at every relevant step across the full clinical timeline.
- *Correct Final Decision (22 cases)* these cases include intermediate errors during the analysis of the patient’s clinical history, but the final classification was still correct.
- *Incorrect (23 cases)* in these cases, the final prediction was incorrect, which could be due to one or more critical errors made during intermediate steps.

The combined total of fully and partially correct predictions indicates that the model is often able to reach the correct conclusion, even when not all intermediate predictions are perfect. This supports the idea that task decomposition can be an effective strategy, provided that key decision points are handled accurately.

**Fig. 11:**
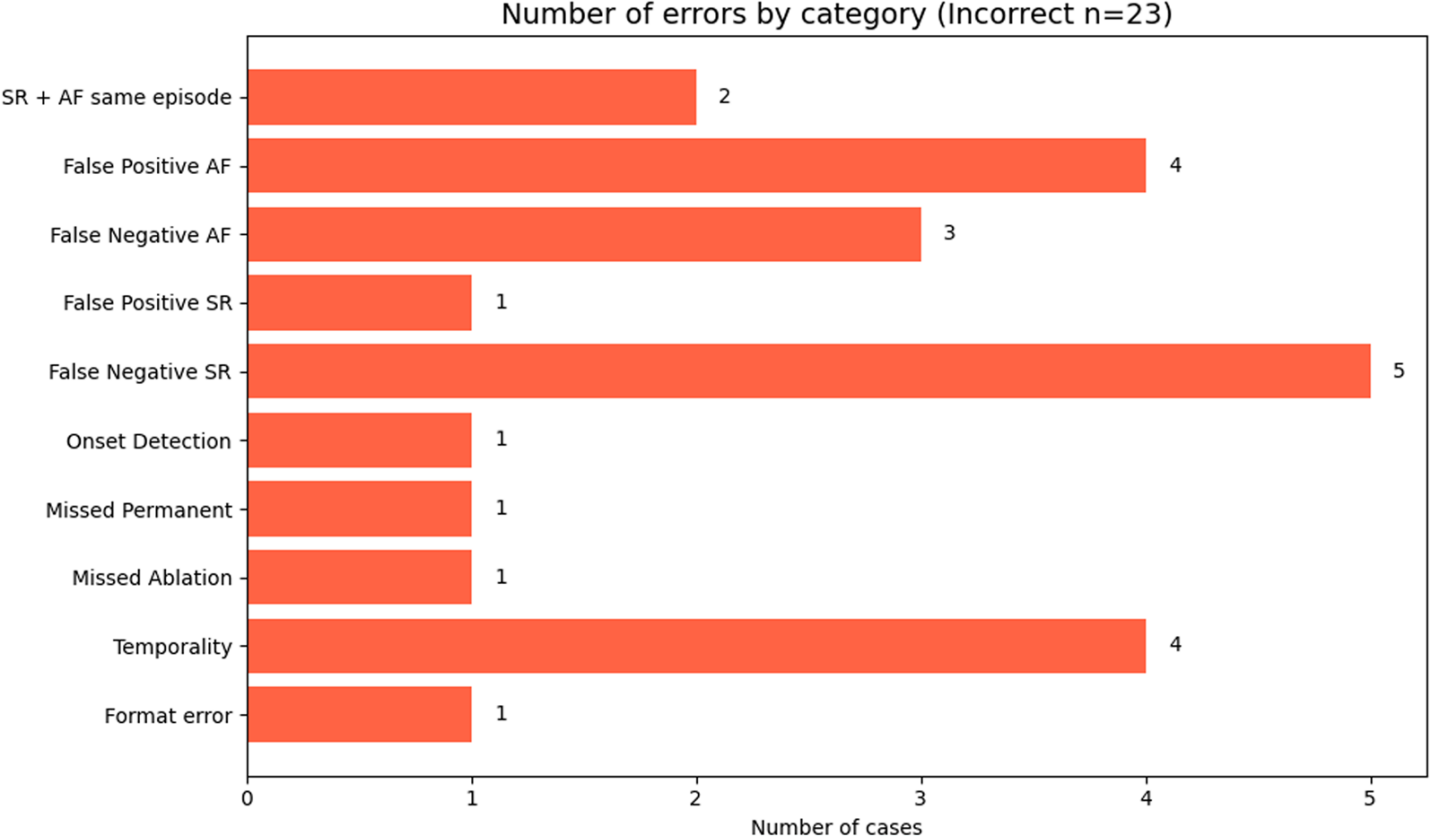
Error analysis for the *Incorrect* category. Each bar represents a specific type of error and the number of times it was made by the model. More detailed description of the errors available in “Appendix 3”

**Fig. 12:**
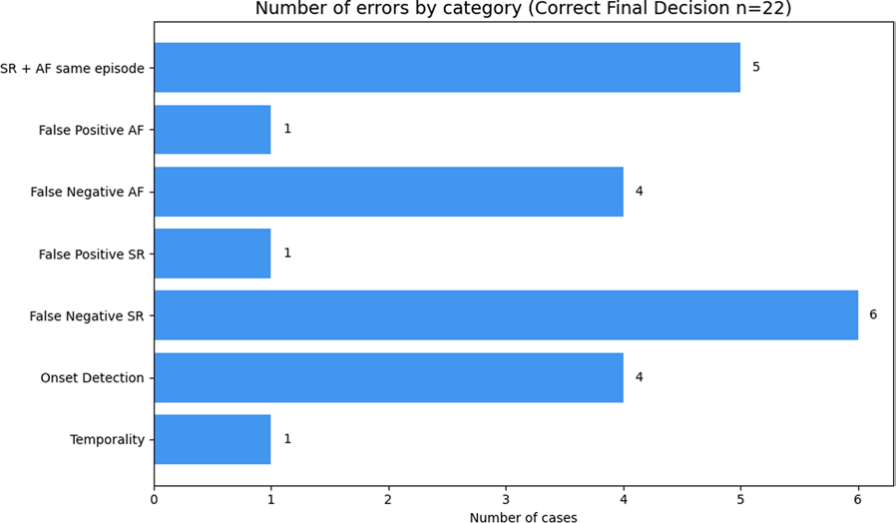
Error analysis for the *Correct Final Decision* category. Each bar represents a specific type of error and the number of times it was made by the model. A more detailed description of the errors is available in “Appendix 3”

A more detailed analysis of the errors made in both the incorrect cases (see Fig. 11) and the partially correct cases (see Fig. 12) reveals key patterns in the model’s behavior. The majority of the errors stem from the second step of the classification process, where AF progression is identified. In particular, many of these misclassifications are linked to difficulties in detecting sinus rhythm (SR), with frequent occurrences of false negatives and confusion between SR and AF within the same clinical episode.

This pattern may help explain the relatively lower performance observed in the *Chronology-Guided* approach, as even small inaccuracies in sinus rhythm identification can propagate and affect the final progression classification. The presence of errors like SR + AF same episode and False Negative SR highlights the ambiguity the model faces in differentiating between arrhythmia progression and recovery within overlapping or sequential events.

While some of these errors could potentially be addressed through further prompt refinement, we argue that continuously adjusting the prompt based on error analysis closely resembles a rule-based approach—where all possible scenarios are manually accounted for. This contradicts the intended flexibility and adaptability that LLMs are designed to offer. In our case, prompt engineering was already carried out during development, and overly tailoring the prompt to specific patterns observed in the results may not only reduce generalizability but also introduce new types of errors.

Furthermore, the incorrect cases reveal additional, less frequent errors such as temporality issues, formatting inconsistencies, and misinterpretations of terms like ‘*permanent*’ or clinical events such as ablation. However, these appear to be isolated instances rather than systematic failures, and therefore do not represent significant limitations of the overall approach.

***Temporality analysis***

**Fig. 13:**
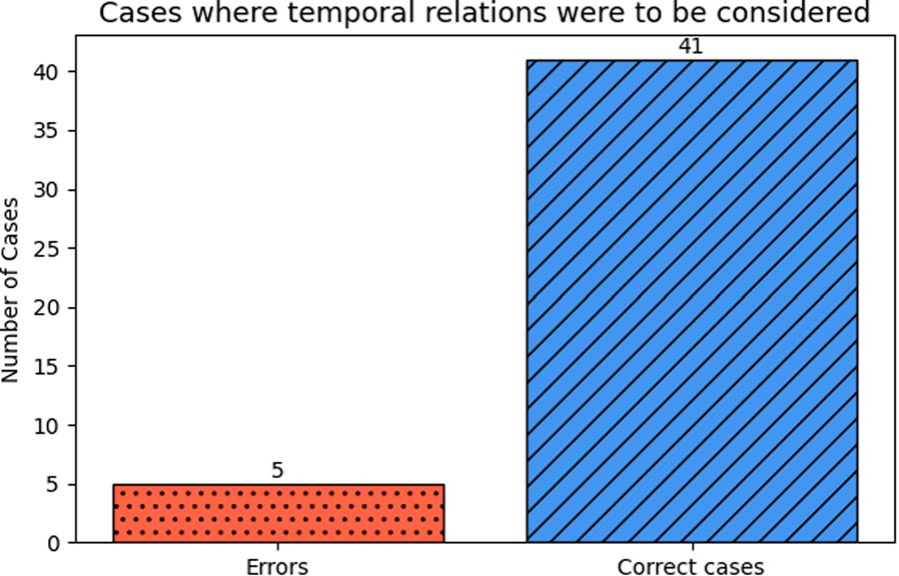
Error analysis in temporal reasoning tasks. Red (dotted) bars indicate cases where the temporal relation (between disease debut and current report) was ignored, while blue (scratched) bars represent correct handling of these temporal dependencies

A closer examination of the cases in which temporal relationships influenced the final decision—such as electrocardiogram findings indicating AF episode either before 1 month or after 2 years from the debut—revealed that, in most instances, the model handled these scenarios correctly (see Fig. 13). This suggests that, when temporal constraints are clearly defined and explicitly presented, the model is generally capable of applying them appropriately, reinforcing the idea that temporality is not a fundamental limitation in this task.

***Uncertainty analysis***

We performed an uncertainty analysis to assess model confidence. Incorporating this type of analysis is essential, as it goes beyond standard accuracy metrics by providing insight into the confidence structure of the model, highlighting systematic sources of uncertainty, and helping to identify borderline cases.

Since greedy decoding only returns the most likely prediction without information about alternatives, we estimated the conditional log-likelihood of all possible labels. This allowed us to quantify uncertainty in the model’s decisions. Our working hypothesis was that incorrect predictions would exhibit smaller probability differences between classes (indicating greater ambiguity) whereas correct predictions would display clearer separation.

To test this, we calculated the log-likelihood difference between the predicted label and the second most probable label. The mean difference was **3.98** (±0.95) for correctly classified patients and **3.73** (±1.00) for misclassified patients, supporting our hypothesis that correct predictions are generally associated with higher confidence and clearer separation.

Because the final decision is composed of multiple steps, a single misclassification can lead to an overall incorrect outcome even if all other steps are correct. To account for this, we computed the confidence difference specifically for the misclassified steps, obtaining a mean value of **3.64**.

Interestingly, the misclassifications with the highest confidence were primarily related to temporality issues. These cases can often be identified when both the debut and progression dates are extracted within less than 1 month of each other. We therefore recommend a manual review of such cases, as well as of instances where the log-likelihood difference is particularly small (e.g., below 3), since these reflect low-confidence model decisions.

To further assess robustness, we designed a set of adversarial examples intended to mislead the model:

- ***Negations*** the presence of negative clauses in AF mentions can mislead the model, particularly when it relies on keyword detection rather than interpreting the full semantic meaning of the sentence. These adversarial cases were manually constructed to assess this vulnerability.
- ***Other arrhythmias*** mentions of arrhythmias other than AF and its synonyms may confuse the model if it lacks the medical knowledge required to distinguish between different conditions. These examples were extracted from real clinical reports.
- ***Two contrary ECGs in the same report*** the presence of two ECGs within the same report (one indicating a new episode of AF and another reporting a return to sinus rhythm) may mislead the model, as shown in the error analysis in Figs. 11 and  12. These adversarial cases were also sourced from real reports.

The adversarial experiments yielded the following results:

- For the **10** ***negation*** **examples**, the model correctly classified 8. The 2 misclassified cases had mean log-likelihood differences of 1.15 and 0.23, reflecting very low confidence.
- For the **20 examples of** ***other arrhythmias***, the model achieved an accuracy of 70%, with the most frequent error being a prediction of $-1$ (Unknown) instead of 0 (Non-Progression). Notably, this type of error is less critical than confusing 1 with 0 or vice versa.
- The **24** ***two contrary ECG*** **cases** were the most challenging, with only 50% accuracy. This appears to stem from the prompt not explicitly requiring that a return to sinus rhythm must occur without any AF episode in the same report. Refining the prompt design for such cases could help mitigate these errors.

### Results for HF decompensation

The *gemma-3-12b-it* model, using the adapted Onset-Guided strategy for the HF decompensation task, achieved an accuracy of 0.72 with the Spanish prompt and 0.74 with the English prompt.

Although the results obtained for the HF decompensation task are slightly lower than those reported for AF progression, they remain positive. This modest decrease in performance may be related to the greater number of reports per patient in the HF dataset (mean of 7.07 reports), compared to the AF dataset (mean of 5.07 reports). As a result, the likelihood of encountering ambiguous or borderline cases increases. In particular, the Non-Decompensation (0) class—already the most challenging to predict—is more prone to misclassification. This is because a correct prediction of this class requires that all intermediate steps confirm the absence of any new decompensation episode, making it more sensitive to errors at any stage of the process.

These results confirmed the strong performance of the stepwise strategy and demonstrate the generalizability of the approach to other diseases where inclusion criteria depend on distinct clinical episodes documented across multiple reports within specific temporal ranges (for instance, Relapsing-Remitting Multiple Sclerosis or Chronic Obstructive Pulmonary Disease).

## Conclusions

This article presents a comprehensive comparison of various NLP-based techniques for automatic cohort selection in a real-world clinical setting, specifically focused on the progression of AF (which is particularly interesting because it is based heavily on temporal reasoning) and also evaluated in HF decompensation. The study explores two distinct approaches: a rule-based method and a zero-shot LLM strategy that requires no task-specific annotation and training.

Regarding **the first research question (RQ1)**, this study demonstrates the potential of using clinical discharge reports—a universally available resource—for automatic cohort selection. Both the rule-based approach and LLM-based strategies produced promising results, highlighting the viability of unstructured text as a primary data source. Importantly, the introduction of a third class, ‘Unknown’, reflects the realities of clinical documentation, where essential information for determining a patient’s status is not always present in discharge summaries alone. In practice, clinicians often consult additional sources such as electrocardiograms or outpatient notes. Therefore, when relying solely on discharge reports, it is both realistic and necessary to account for cases where key information is absent.

Although the potential of NLP techniques for information retrieval and automatic cohort selection has been demonstrated in numerous studies and benchmark challenges such as the TREC Clinical Trials track, this work extends those efforts by evaluating both LLMs and an adapted rule-based methodology in a substantially more complex and realistic clinical scenario. Unlike prior tasks that typically rely on short, isolated descriptions, our approach processes full-length clinical histories composed of multiple discharge reports per patient—reflecting the longitudinal nature of real-world medical records. Furthermore, tasks like detecting AF progression and HF decompensation are inherently more demanding, as they require accurate interpretation of temporal relationships and integration of multiple clinical events to reach a correct classification. An additional layer of complexity stems from the use of Spanish-language EHRs, demonstrating the capability of these methods to operate in less-resourced linguistic settings. Importantly, the clinical documents used in this study are subject to strict privacy constraints, a common condition in actual healthcare environments that contrasts with the publicly available records used in challenges like TREC, highlighting the practicality and applicability of our approach in real-world settings.

**The second research question (RQ2)** lies at the core of this study, aiming to compare rule-based methods with zero-shot LLM approaches for automatic cohort selection. As shown in Table 5, the rule-based method achieved the highest accuracy and F1-score across all evaluated strategies. However, it is important to note that our approach is not strictly rule-based; it incorporates additional NLP modules—namely, section identification, medical entity recognition (MER), and negation detection—to address key limitations of traditional regular-expression-driven systems. These modules help overcome challenges related to negation handling, ambiguity, and lack of generalizability. This reinforces the notion that rule-based methods alone are insufficient for reliable cohort selection and that more advanced NLP techniques are essential.

Despite its strong performance, the rule-based approach has limited adaptability. The auxiliary modules are tailored to the structure and language of the specific discharge reports used in this study, which may hinder their transferability to other clinical settings or document formats. In contrast, LLMs demonstrated comparable results without requiring any domain-specific preprocessing or training. Among the evaluated strategies, the *Onset-Guided* approach—first identifying the AF debut, followed by detecting progression—proved particularly effective, achieving performance levels close to those of the rule-based system. As a zero-shot method, the LLM-based strategy offers greater generalizability and flexibility. To assess the generalizability of this strategy, we additionally applied it to the HF decompensation scenario. After adapting the prompts to this specific context, the approach once again yielded positive results. Therefore, we can conclude that our solution can be readily applied to new report formats or healthcare systems without significant reconfiguration, making it a more scalable and sustainable solution for real-world applications in cohort selection.

The **third research question (RQ3)** was designed to evaluate the ability of LLMs to process long contexts and reason over temporal relationships. The *Concatenation* strategy, in which all reports were concatenated into a single input, revealed a clear limitation of LLMs in handling lengthy contexts with dense information, resulting in poor performance close to random classification. However, when the task was decomposed into sequential steps—processing each report individually—performance improved significantly. The *Onset-Guided* strategy, in particular, achieved results comparable to rule-based cohort selection. Interestingly, increasing the number of processing steps beyond two introduced additional errors, as demonstrated by the degraded performance of the *Chronology-Guided* strategy (which involved three steps). This suggests that while stepwise decomposition enhances LLM performance, excessive fragmentation may harm accuracy due to error propagation or information loss across steps.

Contrary to our initial hypothesis, temporal reasoning did not pose a major challenge for the best-performing model (*gemma-3-12B-it*) especially when explicit debut dates were provided instead of time intervals between events. As illustrated in Fig. 13, *gemma-3-12B-it* correctly resolved most cases where date-related ambiguities could have led to errors.

Regarding the **fourth research question (RQ4)** addressing the impact model size, domain specificity and commercial nature can have on the performance, this study reveals **several important insights**.

First, larger models do not necessarily outperform smaller ones, as shown by the superior performance of *gemma-3-12B-it* over its 27B counterpart. Second, the language used in the prompt influences model behavior, particularly in instruction following—an area where many models, such as Mistral, show limitations. Finally, while the medical version of LLaMA (Aloe-Beta) generally improves performance, the opposite occurs with Gemma, where the clinical variant (MedGemma) tends to include unwanted reasoning, reducing its effectiveness in tasks requiring concise outputs.

Additionally, the use of Spanish discharge reports in this task demonstrates the capabilities of LLMs in lower-resource languages and highlights their potential for clinical applications beyond English-speaking settings. This is particularly relevant given the scarcity of annotated clinical data in languages other than English.

Importantly, all models used in this research are open-source and run locally, reflecting realistic constraints in clinical environments. In contrast to other studies such as [34, 36, 52], which rely on proprietary GPT-based models, our approach aligns with the strict data privacy requirements that typically govern the use of real medical records, offering a more practical and reproducible solution for healthcare institutions.

Although this study has focused specifically on AF progression and HF decompensation, the methodology presented is adaptable to other conditions that require longitudinal analysis of patient histories.

## Figures and Tables

**Fig. 14:**
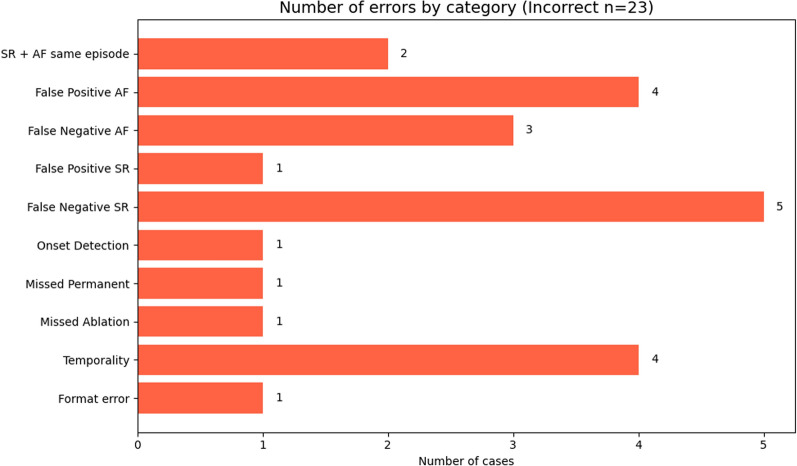
Error analysis for the *Incorrect* category. Each bar represents a specific type of error and the number of times it was made by the model

**Fig. 15:**
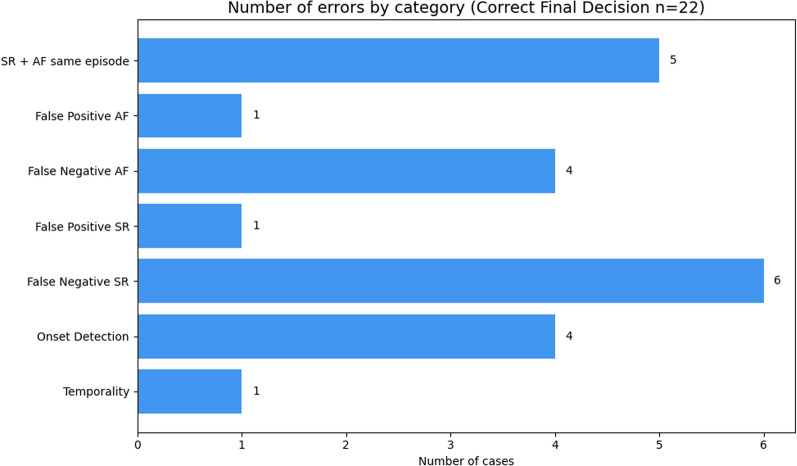
Error analysis for the *Correct Final Decision* category. Each bar represents a specific type of error and the number of times it was made by the model

## Data Availability

The datasets generated and analyzed during the current study are not publicly available due to privacy and confidentiality restrictions.

## Appendix 1: Annotation guidelines

This Appendix presents the annotation guidelines used to label AF progression and HF decompensation in discharge reports.

### AF progression guidelines

**Objective**: Annotate AF progression occurring between 1 month and 2 years after the initial AF episode (debut).

**Clinical question:** *Will this arrhythmia reappear within the next 2 years?*

**Clinical Perspective for the Arrhythmologist**: *Is it advisable to consider ablation at this point?* *Should rhythm control be managed aggressively from now on?*

**Identification of the AF Debut**

1. Report indicating a first AF episode:
  - AF is not mentioned in the patient’s past medical history.
  - AF is reported for the first time under sections such as *Complementary Tests*, *Diagnosis*, or *Clinical Course (Evolution)*.
2. Report mentioning AF in the medical history along with its onset date.

**Identification of AF Progression**: Reports from 1 month to 2 years after debut.

1. Evidence of AF ablation (cryoablation or radiofrequency):
  - The report may correspond to the ablation procedure itself.
  - It may describe the decision to perform ablation.
  - It may mention a past ablation.
2. Evidence of cardioversion performed for AF.
3. Report explicitly mentioning AF recurrence:
  - A new AF diagnosis confirmed by ECG or Holter monitoring, typically documented in *Complementary Tests* or *Clinical Course (Evolution)*.
  - AF recurrence noted in the *Diagnosis* or *Reason for consultation* section.
4. Report stating that the AF type is permanent or chronic.

**Synonyms**:

- ***AF***: FA, ACFA, ACxFA, Fibrilación auricular, Aleteo auricular, Arritmia completa, Fibriloflutter.
- ***Sinusal Rhythm***: Ritmo sinusal, taquicardia sinusal, bradicardia sinusal, RS, Ritmo Basal normal, ECG sin hallazgos / sin anomalías / sin alteraciones, Ritmo auricular normal.

**Labelling Scheme**: For completeness, the labelling process assigns the following values:

- ***Progression*** (1): The patient has a documented recurrence or permanence of AF between 1 month and 2 years after the onset.
- ***Non-progression*** (0): The patient returned to sinus rhythm, with no evidence of AF recurrence or permanence at least 1 day after the onset.
- ***Unknown*** ($-1$): The discharge reports lacked sufficient information to determine the progression status.

### HF progression guidelines

**Objective**: Determine whether a patient experiences HF decompensation within 1 week to 2 years after the initial (debut) episode.

**Clinical question:** *Will the heart failure decompensation happen within the next 2 years?*

**Clinical Perspective for the Arrhythmologist**:

**Identification of the HF Debut**

1. Report indicating a HF episode:
  - HF is not mentioned in the patient’s past medical history.
  - HF is reported for the first time under sections such as *Complementary Tests*, *Diagnosis*, or *Clinical Course (Evolution)*.

**Identification of HF Decompensation**: Reports from 1 week to 2 years after debut.

1. HF is reported under sections such as *Complementary Tests*, *Diagnosis*, or *Clinical Course (Evolution)*

**Synonyms**:

- ***HF***: Insuficiencia cardíaca, IC, Insuficiencia cardíaca congestiva, ICC, falla cardíaca, descompensación hidrópica, congestión pulmonar, fallo derecho, fallo izquierdo.

**Labelling Scheme**: For completeness, the labelling process assigns the following values:

- **Decompensation** (1): The patient has a documented initial episode of HF, followed by a documented episode of HF decompensation occurring between 1 week and 2 years after the debut.
- **Non-Decompensation** (0): The patient has a documented initial episode of HF but no subsequent HF episodes reported.
- **No-debut** ($-1$): The patient has no documented initial episode of HF in any report.

## Appendix 2: Prompts

This section presents the final prompt used for each strategy. All prompts were initially refined using the development set and subsequently evaluated on the test set. To assess the impact of language on model performance, each prompt was also translated into Spanish. A zero-shot approach was selected, as the length of discharge reports, combined with the context limitations of current LLMs, made the inclusion of examples within the same prompt impractical.

### Concatenation strategy

**System**: You are a system that analyzes the clinical reports in Spanish regarding a patient’s history of atrial fibrillation to determine whether a recurrence has occurred. Definitions:

- ‘Yes’ is defined as any of the following:
  - A new episode of AF between 1 month and 2 years after the first documented AF episode.
  - Diagnosis of permanent or chronic AF.
  - A cardioversion or ablation performed during that period.
- ‘No’ is defined as the patient returning to sinus rhythm at least one day after the debut episode.
- ‘Unknown’ must be used if the information is insufficient to confirm either condition.

Respond with only one of the following values:

- Yes
- No
- Unknown

**User**: Given the following medical reports in Spanish, determine whether the patient has experienced a recurrence of atrial fibrillation. DO NOT INCLUDE EXPLANATIONS, respond with only one of the following values:

- Yes
- No
- Unknown

Reports: reports

**Assistant**: Label:

### Summarization strategy

#### Summary prompt

**System**: Your task is to analyze a Spanish medical report and a previous summary (if provided) and extract only the relevant information about:

- First episode of Atrial Fibrillation.
- ECG results, including the date of each ECG.
- Cardioversions and their dates.
- Ablations and their dates.

Combine the extracted information with the previous summary, ensuring:

1. No repeated information (except for ECGs, which may appear multiple times if documented in different reports).
2. The date of the current report is included for context.
3. The output is a concise, unified summary in Spanish, preserving only the key details.

Respond with only one of the following values:

- Yes
- No
- Unknown

**User**: Given the following Spanish medical report and a previous summary (if any), extract the relevant details about Atrial Fibrillation recurrence, ECGs, and procedures as specified. Merge them into a single summary in Spanish, avoiding duplicates (except for ECGs).

Current report: report

Previous summary: previous

**Assistant**: Summary:

#### AF progression detection prompt

**System**: You are a system that analyzes a clinical summary in Spanish regarding a patient’s history of atrial fibrillation (AF) to determine whether a recurrence has occurred. Definitions:

- ‘Yes’ is defined as any of the following:
  - A new episode of AF between 1 month and 2 years after the first documented AF episode.
  - Diagnosis of permanent or chronic AF.
  - A cardioversion or ablation performed during that period.
- ‘No’ is defined as the patient returning to sinus rhythm indicated by an electrocardiogram (ECG) showing ‘RS’ or ‘sinusal’.
- ‘Unknown’ must be used when there is no clear evidence of recurrence nor evidence of non-recurrence.

Respond with only one of the following labels:

- Yes
- No
- Unknown

**User**: Given the following summary of medical reports in Spanish, determine whether the patient experienced a recurrence of atrial fibrillation. Respond with only one of the following values, DO NOT INCLUDE EXPLANATIONS:

- Yes
- No
- Unknown

Summary: summary

**Assistant**: Label:

### Onset-guided strategy

#### Onset detection prompt

**System**: Detect if this Spanish medical report refers to an Atrial Fibrillation (AF) Debut. An AF debut is defined as the first episode of Atrial Fibrillation of a patient. There must not be any previous episode in the past medical history and there must be a new episode in the diagnosis complementary tests or evolution.

Some concepts referring to Atrial Fibrillation in Spanish are: ‘fibrilación auricular’, ‘FA’, ‘ACxFA’, ‘fibriloflutter’, ‘aleteo auricular’ or ‘arritmia completa’. Do not confuse it with ‘flutter’ or ‘flutter auricular’.

**User**: Given a discharge report in Spanish detect if it contains an Atrial Fibrillation debut. Just Answer ‘Yes’ or ‘No’. DO NOT INCLUDE EXPLANATIONS.

Report: report

**Assistant**: Label:

#### AF progression detection prompt

**System**: Given a medical report in Spanish regarding a hospital admission and the Atrial Fibrillation (AF) debut date detect if an AF recurrence has occur. Definitions:

- ‘Yes’ is defined as any of the following:
  - A new episode of AF between 1 month and 2 years after the first documented AF episode.
  - Diagnosis of permanent or chronic AF.
  - A cardioversion or ablation performed during that period.
- ‘No’ is defined as the patient returning to sinus rhythm indicated by an electrocardiogram (ECG) showing ‘RS’ or ‘sinusal’.
- ‘Unknown’ must be used when there is no clear evidence of recurrence nor evidence of non-recurrence.

Respond with only one of the following labels:

- Yes
- No
- Unknown

**User**: Given the following medical report and the date of the atrial fibrillation debut determine whether the patient experienced a recurrence of atrial fibrillation. Respond with only one of the following labels, DO NOT INCLUDE EXPLANATIONS:

- Yes
- No
- Unknown

Debut date: debutdate

Report: report

**Assistant**: Label:

### Chronology-guided strategy

#### Onset detection prompt

The onset detection prompt is the same presented in the Onset-Guided strategy.

#### Sinus rhythm detection prompt

**System**: You are a system that analyzes a Spanish medical report from a hospital admission to determine whether the patient has returned to sinus rhythm. A return to sinus rhythm is confirmed if the electrocardiogram mentions ‘RS’, ‘sinusal’ or similars.

- Respond ‘Yes’ if the report confirms the return to sinus rhythm and there is no mention of a diagnosis of Atrial Fibrillation.
- Respond ‘No’ if there is no clear indication of sinus rhythm or if Atrial Fibrillation is diagnosed in the report.

Respond with only one word: ‘Yes’ or ‘No’.

**User**: Given the following Spanish medical report, determine whether the patient has returned to sinus rhythm. Return only ‘Yes’ or ‘No’.

Report: report

**Assistant**: Label:

#### AF progression detection prompt

**System**: Given a medical report in Spanish related to a hospital admission, return:

Definitions:

- ‘Yes’ is defined as any of the following:
  - A new episode of AF or atrial arrhythmias.
  - Diagnosis of permanent or chronic AF.
  - A cardioversion or ablation performed during that period.
- ‘No’ if the patient has returned to sinus rhythm (SR) or shows an electrocardiogram (ECG) without abnormalities or arrhythmias.
- ‘Unknown’ must be used when no ECG has been performed and there is no clear evidence

Respond with only one of the following labels:

- Yes
- No
- Unknown

**User**: Given the following medical report, determine whether the patient has experienced a recurrence of atrial fibrillation (AF). DO NOT INCLUDE EXPLANATIONS, respond with only one of the following values:

- Yes
- No
- Unknown

Report: report

**Assistant**: Label:

### HF decompensation

#### HF debut prompt

**System**: Detect if this Spanish medical report refers to a Heart Failure (HF) debut. An HF debut is defined as the first episode of Heart Failure of a patient. There must not be any previous episode in the past medical history and there must be a new episode in the diagnosis, complementary tests or evolution.

Some of the most common synonyms are: ‘IC’, ‘ICC’, ‘insuficiencia cardíaca’, ‘falla cardíaca’.

The possible answers are:

- ‘Yes’ if the report refers to a HF debut.
- ‘No’ if the report does not include a HF diagnosis.

Just answer ‘Yes’, or ‘No’. DO NOT INCLUDE EXPLANATIONS.

**User**: Report: report

**Assistant**: Label:

#### HF decompensation prompt

**System**: You will receive a Spanish medical report related to a hospital admission and the date of heart failure (HF) onset. Determine if there is a new episode of HF in the report.

A new episode occurs only if HF is mentioned in the report. Mentions of HF only in past medical history do not count. If the report explicitly states it is the first episode of HF, then it is not a new episode.

Some of the most common synonyms are: ‘IC’, ‘ICC’, ‘insuficiencia cardíaca’, ‘falla cardíaca’.

Return:

- ‘Yes’ if there is a new episode of HF.
- ‘No’ if there is not.

Answer only with ‘Yes’ or ‘No’. DO NOT INCLUDE EXPLANATIONS.

**User**:

Report: report

Debut date: debutdate

**Assistant**: Label:

## Appendix 3: Analysis of errors

The error analysis conducted for the *gemma-12b-it* along with the *onset-guided* strategy, showed the most common errors for both the ‘Incorrect’ and ‘Correct Final Decision’ scenarios (see Figs. 14 and  15). The following categories summarize the types of errors observed:

- **SR + AF in the Same Report**: The discharge report contains multiple ECG results—one indicating AF and another indicating a return to sinus rhythm (SR). This often leads to a false positive detection of SR.
- **False Positive AF**: The model incorrectly identifies an AF episode that is not actually present in the report.
- **False Negative AF**: The model fails to detect an AF episode that is documented in the report.
- **False Positive SR**: The model incorrectly identifies a return to sinus rhythm when no such evidence exists.
- **False Negative SR**: The model does not recognize a return to sinus rhythm that is described in the report.
- **Onset Detection Error**: The initial AF episode (onset) is not correctly identified by the model.
- **Permanent AF Misclassification**: The model fails to identify a diagnosis of permanent AF as a valid progression case.
- **Missed Ablation**: An ablation procedure relevant to AF progression is not detected.
- **Temporality Error**: The model fails to correctly apply the required time window (1 month to 2 years after the debut) when classifying AF progression.
- **Format Error**: The model returns an output that does not match one of the expected labels: ‘Yes’, ‘No’, or ‘Unknown’.

Some of the most frequent errors–particularly false negative* and “SR + AF in the same report”—might be reduced through additional prompt-engineering or by supplying more explicit examples. Yet constantly refining the prompt in response to every new error risks over-fitting the model to idiosyncratic patterns and may introduce fresh mistakes elsewhere. At that point the workflow begins to resemble a rule-based system: the prompt must enumerate an ever-growing list of edge cases, sacrificing the very generalizability that makes LLMs attractive in the first place.

## Footnotes

1 https://icd.who.int/en/.

2 https://classbrowser.nhs.uk/ref_books/OPCS-4.10_NCCS-2024.pdf.

3 https://www.snomed.org/.

4 In this work, AF progression is defined to include all forms of atrial fibrillation–paroxysmal, persistent, and permanent. For paroxysmal AF, progression refers to the recurrence of the arrhythmia, while for persistent and permanent AF, it is defined as the continued presence of the arrhythmia. In both cases, progression must occur within the time window of 1 month to 2 years following the onset (debut) episode.

5 We define AF onset as the patient’s first documented episode of AF.
